# The immune role of the intestinal microbiome in knee osteoarthritis: a review of the possible mechanisms and therapies

**DOI:** 10.3389/fimmu.2023.1168818

**Published:** 2023-06-14

**Authors:** Chang Sun, Xing Zhou, Ting Guo, Jia Meng

**Affiliations:** Department of Orthopedics, Nanjing Jinling Hospital, Affiliated Hospital of Medical School, Nanjing University, Nanjing, China

**Keywords:** gut microbiota, osteoarthritis, gut barrier, adaptive immunity, innate immunity, gut microbiota modulation

## Abstract

Osteoarthritis (OA) is a chronic degenerative joint disease characterized by cartilage damage and synovial inflammation and carries an enormous public health and economic burden. It is crucial to uncover the potential mechanisms of OA pathogenesis to develop new targets for OA treatment. In recent years, the pathogenic role of the gut microbiota in OA has been well recognized. Gut microbiota dysbiosis can break host-gut microbe equilibrium, trigger host immune responses and activate the “gut-joint axis”, which aggravates OA. However, although the role of the gut microbiota in OA is well known, the mechanisms modulating the interactions between the gut microbiota and host immunity remain unclear. This review summarizes research on the gut microbiota and the involved immune cells in OA and interprets the potential mechanisms for the interactions between the gut microbiota and host immune responses from four aspects: gut barrier, innate immunity, adaptive immunity and gut microbiota modulation. Future research should focus on the specific pathogen or the specific changes in the gut microbiota composition to identify the related signaling pathways involved in the pathogenesis of OA. In addition, future studies should include more novel interventions on immune cell modifications and gene regulation of specific gut microbiota related to OA to validate the application of gut microbiota modulation in the onset of OA.

## 1 Introduction

Osteoarthritis (OA) is characterized by degenerative joint damage and synovial inflammation and is a leading cause of disability in elderly individuals. It has been reported that over 22.7 million individuals suffer from arthritis-induced activity limitations and 300 million people worldwide are diagnosed with OA (1, 2). OA can be classified as primary and secondary (such as trauma, surgery on the joint structures and abnormal joints at birth) and usually affects one or several diarthrodial joints, including small joints (e.g., interphalangeal joints) and large joints (e.g., hip joint and knee joint) (3). Previously, biomechanical disorders of the joint and overloading of joints were identified as the causes of OA (4); however, with a growing understanding of OA, it is now widely believed that OA is a much more complex disease with metabolic and inflammatory factor involvement (5). Systemic inflammation and local inflammation of the synovium play crucial roles in the pathogenesis of OA. It has been hypothesized that degraded cartilage can lead to the generation of inflammatory cytokines and metalloproteases (6). Some studies have found a range of immune cells from the synovium in OA patients, including B cells, T cells, lymphoid follicles, granulocytes and plasma cells (7, 8). Moreover, Nedunchezhiyan U et al. proposed a central effect of the innate/adaptive immune response in the pathogenesis of OA (9). To date, researchers have identified several risk factors for OA, such as obesity, estrogen, age, sex, diet, metabolic syndrome, genetics, inflammation, mechanical loading, the oral microbiome and the gut microbiome (10, 11). Most OA patients receive pain-relieving management or joint replacement for end-stage OA with no disease-modifying anti-osteoarthritic drug for OA. Hence, identifying a specific disease-modifying treatment target is imperative.

The gut microbiota comprises multiple microorganisms, including bacteria, yeast, viruses, phages, parasites, and archaea, and due to its important endocrine and immune functions in pathophysiological modulation, the intestinal microbiome has been considered to be a “vital organ” (12). The gut microbiota is involved in many biological functions, including the digestion of food, energetic metabolism, regulation of the immune system, influence of the mucosal barrier and generation of bioactive agents (short-chain fatty acids (SCFAs), estrogen, and serotonin) (13). In clinical studies, Boer et al. reported that the alpha diversity of the gut microbiota was significantly reduced in individuals with OA; however, after adjustment for BMI, the significance was lost (14). The dominant species in the gut microbiome were Bacteroidetes (12.5%) and Firmicutes (77.8%), and plenty of streptococci were linked to worsening levels of pain and local inflammatory responses in the knee joint of OA patients, indicating that the gut microbiota can be a possible starting point for managing knee pain in OA. Bacteroidetes are believed to be associated with anti-inflammatory effects while Firmicutes are supposed to be linked to proinflammatory effects, and the Bacteroidetes/Firmicutes ratio was significantly decreased in OA patients (15). Dysbiosis of the gut microbiome can cause a reduction in the abundance of Bacteroidetes and an increase in that of Firmicutes in OA subjects, which shifts the Bacteroidetes/Firmicutes ratio (15, 16). In addition to the microbiome niches in the traditional gut, Zhao and his colleagues detected bacterial nucleic acids present in the synovium and synovial fluids from subjects with knee OA, which revealed potential correlations with the degree of OA (17). Huang et al. showed that the severity of knee joint damage and synovial inflammation in subjects with OA was positively linked to high levels of lipopolysaccharide (LPS) and the production of intestinal bacteria, and LPS could induce inflammation and damage in joints through macrophage activation and damage-associated molecular patterns (DAMPs), which is named the “two-hit” theory (18, 19). Moreover, Huang et al. showed that mice administered fecal samples from subjects with metabolic syndrome and knee OA exhibited worse OA severity, higher mean circulatory concentrations of inflammatory factors (interleukin-6 (IL-6), IL-1β and macrophage inflammatory protein-1α) and increased intestinal permeability (20), which indicated dysbiosis of the intestinal microbiome played a crucial role in the pathogenesis of OA.

As mentioned above, more studies have focused on the role of the gut microbiota in OA. Many possible mechanisms of the gut microbiota and OA, including low-grade inflammation, metabolic changes and immune modulation, have been proposed. The gut microbiota interacts with risk factors for OA, including obesity, estrogen, age, sex, diet, metabolic syndrome, genetics, inflammation, mechanical loading and exercise (10, 21). Liu et al. reviewed that the gut microbiota, gut microbiota-related components and corresponding metabolites interacted with OA by activating local and systemic innate immune responses (22). Dysbiosis of the intestinal flora can modulate the differentiation of primitive CD4+ T cells into effector T cells or Treg cells, which is crucial for immune homeostasis and joint inflammation (23). Collectively, the gut microbiota may affect the pathogenesis of OA through the immune system. However, the potential mechanisms of the relationship between innate/adaptive immunity and the gut microbiota in knee OA are uncertain. Hence, this paper will summarize the effect of the intestinal microbiota on the intestinal barrier, the immunomodulation of the intestinal microbiota on OA and intestinal microbiota modulation therapy for OA. We hope that the immunomodulation of the gut microbiota in OA patients will be a novel target for OA management in the future.

### 2 The gut microbiota influences the gut barrier

**Figure 1:**
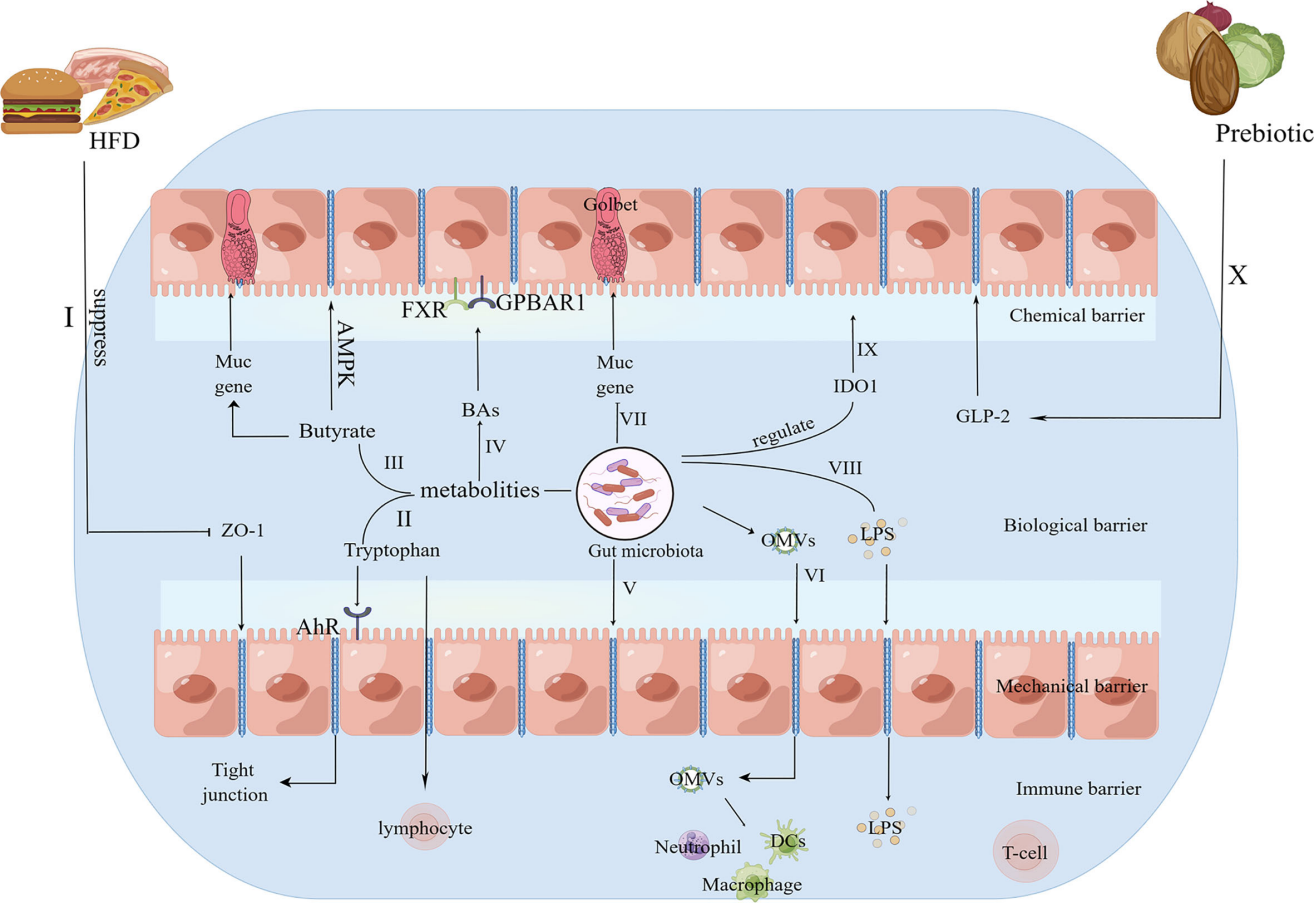
The potential mechanisms for the role of the gut microbiota in the gut barrier. I.HFD consumption suppresses the expression of the tight junction protein ZO-1 to increase intestinal permeability (39, 40). II. AhR, a ligand for tryptophan metabolism, acts on epithelial renewal, barrier integrity, and intraepithelial lymphocytes (41). III. Butyrate specifically modulates MUC gene expression in intestinal epithelial goblet cells to affect the mucus layer (42) and enhances the intestinal barrier by facilitating tight junction assembly via AMPK (43). IV. BAs could protect the gut barrier via the interactions of FXR, GPBAR1 and the gut microbiota (44). V. The gut microbiota can disrupt tight junctions (33). VI. OMVs can disrupt tight junctions, interact with intestinal epithelial cells and enhance the transportation of OMVs as well as their contents of bacterial virulence factors into the submucosa. As a result, OMVs can directly interact with macrophages, neutrophils and DCs in the submucosa (34). VII. The gut microbiota inhibits the expression of the MUC gene (33). VIII. LPS produced by the gut microbiota can enter the systemic circulation via tight junctions (39, 40). IX. The gut microbiota regulates IDO1 activity to exhibit a mucosal protective effect (41). X. Prebiotics improve tight-junction integrity via GLP-2 (35). HFD, high fat diet; ZO-1, zonula occludens 1; AhR, aryl hydrocarbon receptor; MUC, mucin; AMPK, AMP-activated protein kinase; FXR, farnesoid X receptor; GPBAR1, G protein-coupled bile acid receptor 1; OMVs, outer membrane vesicles; LPS, lipopolysaccharide; IDO1, indoleamine 2,3-dioxygenase 1; GLP-2, glucagon-like peptide-2.

The gut barrier has selective permeability for nutrients, water and electrolytes, while preventing harmful substances from transiting through the gut mucus membrane into other tissues, related organs and the circulatory system. The chemical barrier, biological barrier, mechanical barrier and immune barrier form the gut barrier. Bacterial membranes, tight junctions and intestinal epithelial cells form the mechanical barrier, so deleterious entities, such as microbes, intestinal antigens, and intestinal proinflammatory cytokines, are prevented from entering the blood circulation (24). The chemical barrier is the mucus layer adherent to the intestinal epithelium, and mucins, a kind of high-molecular-weight glycoprotein, are the main components of the mucus layer, which is essential for gut permeability by preventing immediate contact of large particles and microorganisms with the intestinal epithelium (25). The immune barrier consists of gut-associated lymphoid tissues, such as intraepithelial lymphocytes and mesenteric lymph nodes, which prime innate immunity and adaptive immunity in response to the gut microbiota (25–27). The biological barrier refers to the microspatial construction between the gut microbiota and the gut epithelium (27). Gut permeability refers to the nonmediated gut pathway for medium-sized hydrophilic compounds, which acts via a concentration gradient without the participation of the carrier system (28). Solutes pass through the gut barrier through the paracellular pathway or transcellular pathway. The paracellular pathway is defined as the pathway between cells via interstitial spaces and tight junctions, and medium-sized hydrophilic compounds instead of protein-sized molecules are allowed to pass, thus modulating the transportation of gut microbiota products and other polymers (29, 30). The transcellular pathway represents the passive transportation of energy-dependent uptake and lipid-soluble, small hydrophilic molecules via the intestinal epithelium, while harmful substances, such as intestinal microflora products and inflammatory factors, cannot pass through the gut epithelium (31, 32). Various factors, including cytokines, nutritional factors, and local immune tissues, modulate gut permeability, and the gut microbiota is associated with gut permeability via the disruption of tight junction competency and the inhibition of mucin (MUC) gene expression (33). Morbific and symbiotic gram-negative bacteria can produce outer membrane vesicles (OMVs), and OMVs can disrupt tight junctions, interact with intestinal epithelial cells and enhance the transportation of OMVs as well as their contents of bacterial virulence factors into the submucosa. As a result, OMVs can directly interact with macrophages, neutrophils and dendritic cells (DCs) in the submucosa (34). The composition of the intestinal microbiome and intestinal permeability can be modulated by prebiotics in a glucagon-like peptide-2 (GLP-2) manner, which could improve tight-junction integrity (35). In addition, the intestinal microflora also works with the host immune system to participate in the pathogenesis and progression of OA (36). Related studies have observed crosstalk between the imbalance in the intestinal flora and OA by a gut-joint axis; as a result, dysbiosis can induce abnormal intestinal mucosal barrier function and increase the permeability of the intestinal barrier, thereby allowing inflammatory factors and microflora products into the circulatory system and inducing the onset and development of OA (37, 38). Therefore, a well-functioning intestinal barrier is essential to slow the progression of OA. The corresponding mechanisms of the effects of the intestinal microbiota on the gut barrier are summarized in **Figure 1**.

Gut microbiota dysbiosis can cause changes in bacterial metabolism products and abnormal nutrient absorption, thus affecting gut permeability to allow bacterial products and inflammatory factors into the systemic circulation. Currently, the most studied categories of metabolism in association with host-microbiota interactions are bile acids (BAs), SCFAs, and metabolites of tryptophan. Dietary fibers are fermented by the anaerobic microflora in the gut with the generation of SCFAs as the end products, which indicates a healthy microbial community (45). When gut microbiota dysbiosis occurs, the categories and components of SCFAs in the intestine will be changed. Butyrate, propionate and acetate are the most common representative SCFAs and are considered to be involved in bone metabolism (46, 47). Fermentation of dietary fiber by the gut microbiota in the colon can generate butyrate, and butyrate provides the primary energy for colonic intestinal bacteria. In addition, butyrate can weaken intestinal permeability to enhance the gut barrier (48, 49). Intestinal epithelial goblet cells deprived of glucose could express MUC genes, which could be specifically modulated by butyrate (42). The diverse effects of butyrate a manner dependent on MUC gene expression could affect the properties and composition of the mucus gel; as a consequence, the protective effects of mucus in the gut barrier can be altered (42). In addition, butyrate can facilitate tight junction assembly by activating AMP-activated protein kinase (AMPK) in Caco-2 cell monolayers, which ultimately enhances the gut barrier (43). A high-fat/high-sucrose diet can induce Lactobacillus spp. and Methanobrevibacter spp. abundance in the intestine and can increase serum LPS levels and inflammatory factor levels in the blood and synovial fluid, thus accelerating OA in rat OA models (50). LPS is mainly produced by gram-negative bacteria, and can cause inflammatory responses. An excess of transportation of LPS into the circulatory system is associated with OA. High-fat diet (HFD) consumption markedly enhances gut permeability and reduces zonula occludens 1 (ZO-1) expression, a gene encoding proteins of tight junctions, which ultimately allows a large amount of LPS into the systemic circulation (39, 40). The concentrations of serum LPS, synovial fluid LPS and serum LPS binding protein (LBP) were positively linked to the severity level of knee osteophytes, and synovial fluid LPS was significantly linked to the reduced gap in the knee joint space, higher total Western Ontario and McMaster Universities Osteoarthritis Index (WOMAC) scores and self-reported pain scores of the knee (18). Interestingly, serum LPS, serum LBP and synovial fluid LPS were positively associated with the enrichment of activated macrophages (18). In an injury-induced mouse OA model, germ-free mice showed reduced LBP and improved progression of OA (48). Taken together, gut microbiota dysbiosis can accelerate the progression of OA in injury-induced animal models via increased LPS release into the circulatory system, whereas SCFAs can protect the gut barrier from enteric dysbacteriosis.

For many microflora, tryptophan is an important biosynthetic precursor (51). Tryptophan metabolism occurs in the gastrointestinal tract following three pathways: (1) the kynurenine pathway via indoleamine 2,3-dioxygenase 1 (IDO1) in both epithelial and immune cells; (2) gut microorganisms directly transforming tryptophan into compounds, such as indole and its derivatives, which are ligands of the aryl hydrocarbon receptor (AhR); and (3) enterochromaffin cells synthesizing 5-hydroxytryptamine (5-HT) via tryptophan hydroxylase 1 (TpH1) (52). The AhR signalling pathway works on various types of immune cells, epithelial cell renewal and barrier integrity, thus maintaining the gut barrier and the homeostasis of intraepithelial lymphocytes (53). The gut microbiota can regulate IDO1 activity, and kynurenine-related products, such as Kna, exhibit mucosal protective and immunoregulatory effects (41). 5-HT acts as a pivotal regulator of gastrointestinal tract secretion and motility. Interestingly, the origin of 5-HT determines the impact of 5-HT in controlling bone formation and bone absorption. Ninety-five percent of 5-HT is produced by duodenum chromaffin cells, and gut-derived 5-HT can inhibit bone formation by attenuating the proliferation of osteoblasts through the activation of preosteoblast receptors (54, 55). The gut microbiota can modulate gut 5-HT production through SCFAs, which increase the synthesis of gut-derived 5-HT (56, 57). In contrast, 5-HT produced in the brain can facilitate bone formation via various mechanisms (55). In summary, tryptophan plays a vital role in maintaining the balance between intestinal microflora homeostasis and intestinal immune tolerance.

Cholesterol in the liver can synthesize BAs via two mechanisms. The main mechanism is catalysed via cholesterol-7 α-hydroxylase (CYP7A1), finally generating cholic acid and chenodeoxycholic acid (58). The alternative pathway is accomplished by producing 27-hydroxycholesterol via sterol-27-hydroxylase (CYP27A1), finally generating chenodeoxycholic acid. The gut microbiota can modulate the expression of CYP7A1, CYP7B1, and CYP27A1, thereby influencing BA metabolites (59, 60). On the other hand, BAs can regulate bacterial growth and protect the gut barrier via the interactions of G protein-coupled bile acid receptor 1 (GPBAR1), farnesoid X receptor (FXR) and the gut flora (44). Senescence of chondrocytes is believed to be conducive to the onset of OA. Huang et al. revealed that the bile acid receptor GPBAR1 can play a crucial role in protecting chondrocytes against IL-1β-induced chondrocyte senescence (61). Simon et al. reviewed that BAs can act as determinants of intestinal homeostasis and functional dyspepsia (62). In addition, BAs also influence the components of the intestinal microflora by altering the host’s intestinal immunity and the natural antimicrobial defense of the host in liver diseases (63). Due to the effects of BAs in the intestinal immune system, BAs can influence the gut barrier. In summary, gut microbiota dysbiosis can disrupt the gut barrier, increase intestinal permeability and allow harmful bacterial metabolites into the systemic circulation, thus affecting the onset and development of OA. Therefore, more detailed research on the relationships between the intestinal microflora and OA is essential for subsequent targeted interventions for OA.

## 3 The gut microbiota modulates innate immunity

**Figure 2:**
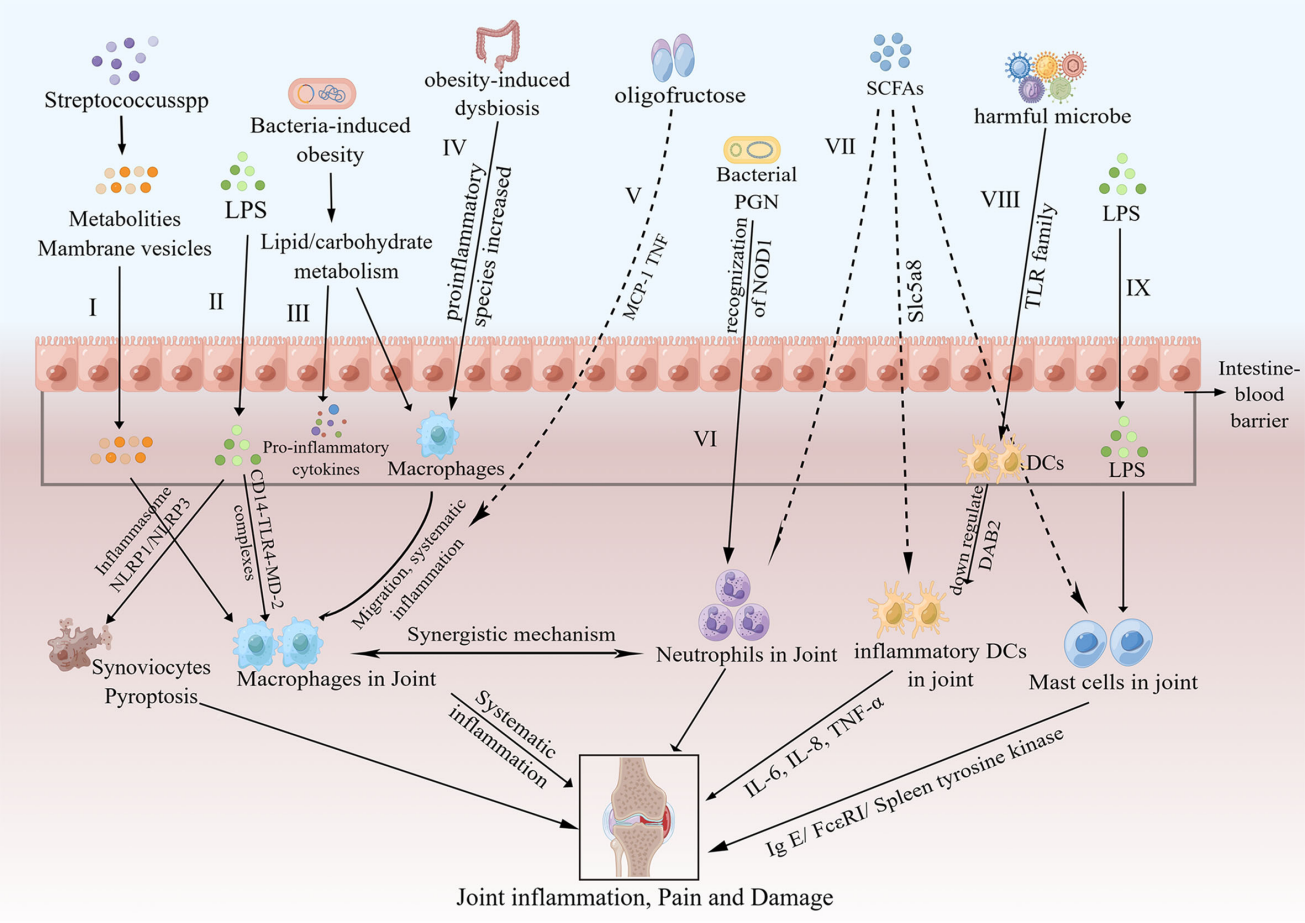
Pathways of the effects of the gut microbiota on OA via innate immunity. I. Metabolites and membrane vesicles produced by Streptococcus species could pass through the intestinal barrier and activate macrophages in the synovial lining, thus causing low-level systemic inflammation, joint inflammation and joint damage (69). II. LPS passes through the intestinal barrier and activates joint macrophages via CD14–TLR4–MD-2 complexes; moreover, LPS induces synoviocyte pyroptosis via either NLRP1 or NLRP3 inflammasomes (19, 70). III. Bacteria-induced obesity could increase lipid/carbohydrate metabolism-related gene expression, which activates macrophages and increases proinflammatory cytokine expression (71). IV. Obesity increases the abundance of key proinflammatory species, affects the migration of macrophages to joints and results in downstream systemic inflammation (72). V. Oligofructose could decrease systematic inflammation by inhibiting MCP-1 and TNF (72). VI. Bacterial PGN enhances neutrophil function via the recognition of NOD1 (73). VII. SCFAs reverse the recruitment of neutrophils (74), inhibit the development of DCs through Slc5a8 by inhibition of HDAC (75) and suppress mast cell degranulation (76, 77). VIII. After exposure to harmful microbes, DCs are switched to inflammatory DCs by downregulation of DAB2 (78) and were stimulated by TLRs to produce more inflammatory cytokines, such as IL-6, IL-8 and TNF-α (79). IX. LPS could activate mast cells (80), and mast cell activation expediates the progression of OA via the IgE/FcϵRI/spleen tyrosine kinase signalling axis (81). LPS, lipopolysaccharide; CD14–TLR4–MD-2 complex, CD14-toll like receptor 4-myeloid differentiation protein-2 complex; NLRP, nod−like receptor protein; MCP-1, monocyte chemotactic protein 1; TNF, tumor necrosis factor; PGN, peptidoglycan; SCFAs, short-chain fatty acids; DCs, dendritic cells; HDAC, histone deacetylases; Slc5a8, Na (+)-coupled monocarboxylate transporter; DAB2, disabled homolog 2; FcϵRI, IgE receptor.

Innate immunity is a primitive mechanism using germline-encoded proteins to recognize pathogens, thereby inducing immune responses. When a pathogen is encountered, the innate immune cell either kills the pathogen or stimulates the adaptive immune response to deal with the pathogen. The activation of the innate immune system plays a crucial role in the pathogenesis and progression of OA by recognizing DAMPs through interactions with pattern-recognition receptors (PRRs) (64, 65). PRRs, which exist on the cell surface, are cytosolic and endosomal receptors consisting of NOD-like receptors, Toll-like receptors (TLRs) and so on (65). PRRs, which widely exist on the outer membrane of macrophages and other immune cells, can identify a large amount of danger signals, similar to gut microbiota metabolites in the innate immune system; as a consequence, the downstream inflammatory signalling pathway is activated (66). Dunn CM et al. detected microbial DNA and the intestinal microbiome at the same time in the knees of OA patients, and they assumed that enteric dysbacteriosis can stimulate the innate immune system to expedite OA progression (19, 67). In addition, Liu et al. showed that the gut microbiota, gut microbiota-related components and corresponding metabolites interacted with OA by activating local and systemic innate immune responses (22). The process by which the innate immune system affects OA includes the following: 1) synovial joint immune cells are activated and generate DAMPs by interactions with invariable PRRs; 2) host responses to DAMPs activate the innate immune response; and 3) rapid-onset inflammatory responses are initiated (68). The innate immune cells consist of macrophages, neutrophils, DCs, natural killer (NK) cells, and mast cells, among others. Next, we will show the regulation of different innate immune cells in the gut microbiota and OA separately (**Figure 2**).

### 3.1 Macrophages in the gut microbiota and OA

In the knee synovium of OA patients, macrophages are the most widespread cell types (82), and are vital components of innate immune cells. Macrophages are a heterogeneous population, and the prominent features of macrophages are the functions of their surface markers and cytokine secretion. Based on the surface marker phenotype, macrophages are divided into “classically activated macrophages (M1)” and “alternatively activated macrophages (M2)” (83). Proinflammatory factors, such as LPS and IFN-γ, can activate M1 macrophages, and once activated, they can generate proinflammatory cytokines including inducible NOS (iNOS), IL-6, IL-1β and tumor necrosis factor-α (TNF-α), so M1 macrophages are thought to be proinflammatory. M2 macrophages, triggered by IL-13 and IL-4, are considered to be inflammatory inhibitors and can generate a large number of anti-inflammatory cytokines consisting of arginase, IL-1 decoy receptor and IL-10, which block the activity of IL-1β and TNF-α (83).

CD14 exists in various immune cells, such as monocytes and macrophages, and interacts with complexes of LPS and LBP (84, 85). LPS initiates proinflammatory innate immune responses via the CD14-LPS-LBP complex and the cd14-TLR4-myeloid differentiation protein-2 (MD-2) complex (86). As a result, the secretion of proinflammatory cytokines, including transforming growth factor-beta (TGF-β), matrix metalloproteinases (MMPs), TNF-α, IL-15, IL-10, IL-8, IL-6, IL-1β and free radicals, is induced (87, 88), which contribute to the secondary inflammatory responses in the joint tissue and exacerbate OA. To date, many researchers have conducted a large number of studies on macrophages and macrophage-produced mediators, and they have found that macrophages and their mediators were significantly associated with inflammatory changes and devastating responses in the synovial membrane and cartilage of OA (82). Huang et al. strongly proposed the role of LPS in the pathogenesis and severity of knee osteophytes, joint space size and pain degree in knee OA patients (18) and concluded that a two-hit theory can account for the potential mechanisms of LPS in OA progression (19). The first hit occurs when LPS activates joint macrophages via CD14–TLR4–MD-2 complexes and the second hit occurs when LPS induces the overall inflammatory response and the destruction of the joint structure due to the coexistence and complementarity mechanism, such as the inflammasome pathway or DAMPs in the form of broken cartilage-matrix molecules (19). In addition, LPS can induce fibroblast-like synoviocyte pyroptosis via either nod−like receptor protein (NLRP) 1 or NLRP3 inflammasomes, which contributes to the progression of OA (70). Taken together, LPS plays a vital role in innate immunity and macrophage-associated inflammatory responses, which are important to the pathogenesis of osteoarthritis.

According to the research by Kraus et al., the number of activated macrophages was significantly associated with pain symptoms in the knee as well as the radiological severity of knee OA. In addition, they reported that macrophages are the cause of OA-related pain at the affected joint site (89). Analogously, Daghestani et al. reported that in the synovium and joint capsule from subjects with knee osteoarthritis, many activated macrophages were detected; furthermore, they demonstrated that activated macrophages were positively associated with the degree of knee pain, decreased joint space and joint destruction in knee OA patients (90). Hsueh et al. found that the main immune cell types in the knee joints of OA patients were macrophages and neutrophils, which contribute to the pathogenesis and deterioration of OA in a synergistic way (91). Among the various risk factors for OA, obesity is widely accepted and can affect OA through the dysbiosis of the gut microbiota, which can increase systemic inflammation (92). According to the study by Schott et al., obesity decreases the amount of beneficial Bifidobacteria and increases the number of key proinflammatory species in the murine gut; as a consequence, the migration of macrophages to knee synovial tissues, the activation of downstream circulatory inflammatory responses and more destruction in the knee cartilage are observed (72). Oligofructose, a kind of nondigestible prebiotic fiber, can repair the imbalance in enteric dysbacteriosis, particularly the abundance of Bifidobacterium pseudolongum, and decrease systemic inflammation by downregulating the expression of TNF and monocyte chemotactic protein 1 (MCP-1) (72). In the Rotterdam Study and a large population-based cohort study, Boer et al. revealed that changes in the gut microbiota, especially a large number of streptococci, are significantly linked to enhanced pain symptoms in the knee. The possible mechanism is that metabolites and membrane vesicles produced by Streptococcus species can pass through the gut barrier and activate synovial macrophages to trigger low-grade systemic inflammation and aggravate joint inflammation and injury (14). Bacteria-induced obesity is known to increase the expression of genes involved in carbohydrate and lipid metabolism (69), thus activating macrophages and the migration of related inflammatory cells to fatty tissues, which release proinflammatory mediators into the blood circulation and aggravate systemic inflammation and the development of osteoarthritis (71). According to the research by Huang et al., fecal microbiota transplantation from subjects with OA and metabolic syndrome accelerates OA in mice, which activates TGF-β signalling pathways to regulate multiple immune cells, such as macrophages, NK cells, DCs, T cells and B cells (20). In summary, macrophages play a vital role in the immune responses between the intestinal microbiome and OA, and more studies need to be conducted to uncover the precise mechanism between macrophages, the intestinal microflora and OA, which may provide a novel target for macrophage immunomodulation in OA treatment.

### 3.2 Neutrophils in the gut microbiota and OA

As the body’s first line of defense against microorganisms, neutrophils play a crucial role in innate immunity (93). Brotfain et al. reported that neutrophils from obese subjects were primed with enhanced chemotactic activity, increased superoxide generation and normal adherence and phagocytosis; therefore, neutrophils may participate in the progression of osteoarthritis with obesity as a crucial risk factor (94). Hsueh et al. showed that the number of neutrophils was the highest in knee synovial fluid with elevated TGF-β and elastase, which are significantly associated with the severity of radiographic knee OA (91). Furthermore, neutrophils and macrophages in OA knee joints act in a synergistic manner during the development and deterioration of OA (91). Kyburz et al. revealed that bacterial peptidoglycan (PGN) can activate synovial fibroblasts via TLR2 and induce MMP and proinflammatory cytokine secretion, thus resulting in joint inflammation and destruction (95). In addition, van der Heijden et al. detected bacterial PGN in knee synovial tissues of individuals suffering from OA (96), where PGN might enhance synovial inflammation. Clarke et al. revealed that PGN, derived from the gut microbiome, systemically primes the innate immune response via the recognition of NOD1, enhancing neutrophil function (73). Interestingly, in mice with no-fiber supplementation for 2 weeks, significant alterations in the intestinal microbiome and significantly enhanced neutrophil-endothelial interactions in the colonic microvasculature were observed by Shen et al. Moreover, supplementation with acetate reversed the recruitment of neutrophils, which indicates the crucial participation of SCFAs in the modulation of neutrophils (74). Taken together, productions and metabolites by the gut microbiota may influence OA progression by interacting with neutrophils. To date, there have been no such studies, and more studies are warranted to uncover the potential relationship between neutrophils, OA and the gut microbiome in the future.

### 3.3 Dendritic cells in the gut microbiota and OA

DCs are antigen-presenting cells derived from monocytes and can recognize and react to pathogen-associated and danger-associated signals, therefore bridging the innate and adaptive immune systems. Mature DCs mainly activate T cells to prime the adaptive immune system. Unlike macrophages, DCs do not have typical surface markers and are divided into plasmacytoid DCs (pDCs) and myeloid DCs (cDCs), and myeloid DCs are composed of myeloid cDC1 and myeloid cDC2 on the basis of cell lineage and correlate with the differential expression of essential transcription factors, such as interferon regulatory factors 8 and 4 (97). Now, a unified classification of mammalian DCs has been introduced, and another type of DC has been defined as monocyte-derived inflammatory DCs, which are distributed in the inflammatory site (98). In healthy subjects, different DC subsets in the intestinal mucosa are present in the tolerogenic form. Once exposed to microbes, DCs are switched to an inflammatory phenotype by downregulation of disabled homolog 2 (DAB2), which is inhibited by the interactions of the TLR ligands TRIF and MyD88 (78). Butyrate and propionate, bacterial fermentation products, can inhibit the development of DCs through the Na (+)-coupled monocarboxylate transporter (Slc5a8) by inhibiting histone deacetylases (HDACs) (75). In addition, Trompette et al. found that propionate can protect against allergic inflammation by modulating DCs, which are dependent on G protein-coupled receptor 41 (99). Engevik et al. showed that Lactobacillus reuteri surface components and metabolites can promote immature DC maturation and enhance anti-inflammatory IL-10 production by DCs (100). At the OA site, DCs are mainly derived from blood mononuclear cells and manifest a proinflammatory phenotype (101). The TLR family, especially TLR4, plays a vital role in the initiation of DCs in osteoarthritis, which expedites the progression of osteoarthritis (78, 79, 102). Nie et al. showed that TLR 1-8 expression was significantly elevated in DCs of OA mice, and TLRs can stimulate DCs to produce more proinflammatory factors, such as TNF-α, IL-6 and IL-8, while inhibition of TLR in DCs reversed the inflammatory response (79). Therefore, the gut microbiota may affect inflammatory DC function, which interacts with TLR family members to promote the pathogenesis of OA. However, more research is urgently needed to verify the interactive mechanism between the gut microbiota, DCs and OA.

### 3.4 NK cells in the gut microbiota and OA

NK cells are CD56+CD3- lymphocytes comprising approximately 15% of all circulating lymphocytes, and can kill microorganisms through death-inducing receptors or the release of soluble molecules, including perforin and granulysin, so NK cells are vital components of the innate immune system. Based on the density of CD56 on the cell surface, NK cells are distributed into two cell subsets: CD56(bright) and CD56(dim) (103). CD56(dim) NK cells, with elevated levels of FC gamma receptor III (CD16) and Ig-like NK receptors, exhibit more natural cytotoxicity (103). In contrast, CD56(bright) NK cells produce more cytokines, show lower natural cytotoxicity and are CD16(dim) or CD16(-) (103). In addition, NK cells can interact with other immune cells to exhibit indirect antibacterial ability. For example, IFN-γ, produced by NK cells, can stimulate macrophages/monocytes and neutrophils to migrate and adhere; as a consequence, the related phagocytosis and oxidative killing effects are activated (104). In addition, IFN-γ can help DCs mature and initiate the generation of cytokines, such as IL-12 and TNF-α (104). NK cells can secrete chemotactic and antimicrobial peptides, such as α-defensins and cathelicidin (LL-37), and LL-37 has been proven to be chemotactic for CD4+ T cells and polymorphonuclear leukocytes, which may participate in the antimicrobial progress of NK cells (105).

Huss et al. found that NK cells contained almost 30% of the CD45+ lymphocytes in the synovium of OA patients and expressed the chemoattractant receptors CCR5 and CXCR3. Compared with blood NK cells, NK cells in the synovium of OA patients show a silent phenotype consistent with post-activated exhaustion, which is impaired by cytokine-stimulated IFN-γ production (106). Jaime et al. found that, in comparison with peripheral blood lymphocytes, most NK cells in the synovial fluid were CD56+CD16(-) NK lymphocytes, which expressed a lower number of cytotoxic mediators (perforin and granzyme B) (107). NKG2D (NK group 2, member D) is a danger sensor and a valid activator of immune responses, and NK cells express NKG2D to recognize and clear infected and transformed cells expressing cognate ligands (108). In addition, NKG2D enhances Th1 and proinflammatory Th17 cell effector functions with high production of proinflammatory cytokines during antigen-induced arthritis (108). In summary, unlike NK cells in the blood, NK cells in OA knee synovial fluid present a quiescent phenotype; however, IFN-γ produced by NK cells can activate neutrophils, macrophages, and DCs. In addition, NKG2D expressed by NK cells can activate T cells, so further investigations are needed to interpret the precise mechanism of NK cells in the gut microbiota and OA.

### 3.5 Mast cells in the gut microbiota and OA

Mast cells act as sentinels in the innate immune system and respond to endogenous danger signals as well as exogenous pathogens rapidly (109). Various factors, such as the IgE receptor FcϵRI, IL-33 and complement receptor C5aR, can induce mast cell degranulation to release preformed mediators, such as proinflammatory lipids, tryptases, histamine, chemokines and cytokines (81). Zhang et al. reported that, in mast cells derived from murine bone marrow, butyrate suppresses FcϵRI-dependent cytokine production, such as IL-6 and TNF-α via HDAC inhibition (76). Similarly, Folkerts et al. documented that propionate and butyrate could suppress the degranulation of human or mouse mast cells with or without IgE mediation, which was associated with HDAC inhibition (77). Further investigation showed that butyrate downregulated the tyrosine kinases BTK, SYK, and LAT by reducing acetylation at the related promoter regions, which are critical transducers of FcϵRI-mediated signals (77). In addition, LPS can activate mature mast cells to produce tryptase, chymase and carboxypeptidase (80). Similarly, Gupta et al. observed that mast cells were activated by LPS once exposed to bacterial infection (110).

It is widely known that mast cells and their mediators are distributed in the synovial fluids and synovial tissues of OA patients (81). In addition, Kulkarni et al. reported that mast cells were differentially distributed in osteophytes and knee synovial fluid, which may further accentuate the inflammatory pathology of OA (111). De Lange-Brokaar et al. revealed that the numbers and degranulation status of mast cells were positively linked to worsening cartilage injury and aggravated synovitis in people with OA, indicating that mast cells contribute to the progression of OA (112). According to Wang et al., mast cell deficiency improved osteoarthritis in mast cell-knockdown mice, and tryptase, a specific product of mast cells, induced inflammation, chondrocyte apoptosis, and cartilage breakdown (81). The possible mechanism of mast cells in the progression of OA is via the IgE/FcϵRI/spleen tyrosine kinase signalling axis (81). Interestingly, Zhao et al. proposed a new synovial tissue pathotype (mast cell-low, mast cell-medium, and mast cell-high) for OA patient joints on the basis of the differential expression of prototypical and distinct mast cell markers, and pharmacologic blockade of histamine activity can reduce the severity and OA-related mediators in a mouse OA model (113). Hence, further investigation into the relationship between mast cells in the gut microbiota and OA may provide a novel direction for OA treatment.

## 4 The gut microbiota modulates adaptive immunity

**Figure 3:**
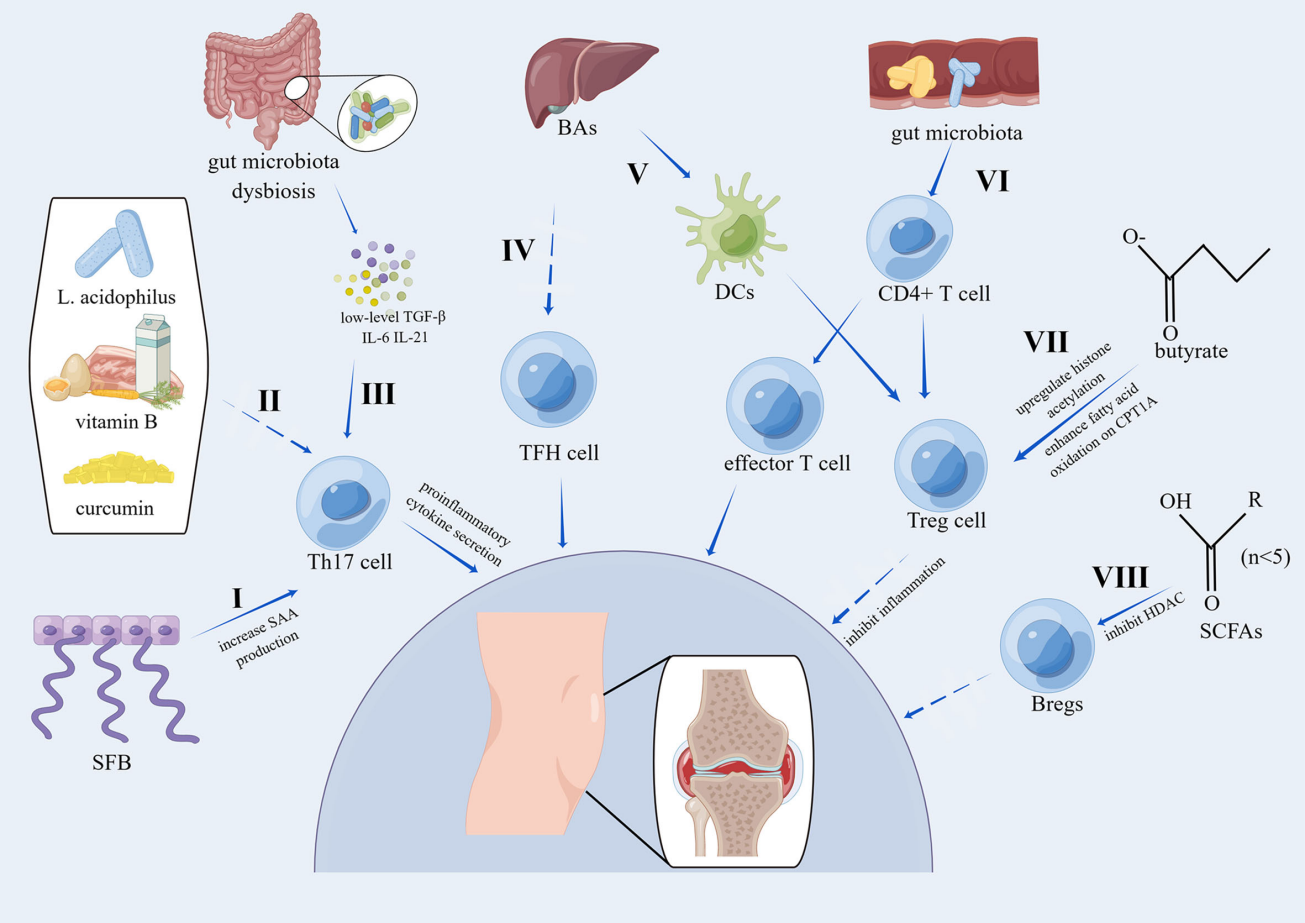
Pathways of the effects of the gut microbiota on OA via adaptive immunity. I. SFB induces the accumulation of Th17 cells by increasing local SAA production, which can act on lamina propria DCs to stimulate the induction of Th17 cells (23). II. The combination of L. acidophilus, vitamin B, and curcumin suppresses Th17 cell differentiation by phosphorylating STAT3 (117). III. Activated Th17 cells can secrete proinflammatory cytokines, and Th17 cells are induced by low levels of TGF-β, IL-6, and IL-21 (118). IV. BAs can inhibit the activation of TFH cells (119). V. 3β-Hydroxydeoxycholic acid, a kind of secondary BA, can induce colonic Treg cells by acting on DCs in a CNS1-dependent manner (120). VI. Gut microbiota dysbiosis modulates the differentiation of CD4+ T cells into Treg cells or effector T cells (23). VII. Treg cells have been proven to inhibit inflammation (121) and are induced by butyrate by upregulating histone acetylation or enhancing fatty acid oxidation in a CPT1A-dependent manner (122). VIII. SCFAs can upregulate Breg cells in a manner dependent on HDAC inhibitory activity (123). SFB, segmented filamentous bacteria; SAA, serum amyloid A; DCs, dendritic cells; BAs, bile acids; CPT1A, carnitine palmitoyl transferase 1A; HDAC, histone deacetylases; STAT3, signal transducer and activator of transcription 3; CNS1, conserved non-coding sequence 1.

As described above, most studies have focused on the effect of innate immunity on the gut microbiota and osteoarthritis, especially on macrophages. Recently, some studies have focused on the effect of adaptive immunity on the intestinal microbiome and OA. Lymphocytes account for 10% of nonadipocyte cells in human adipose tissue and include B cells, T cells, innate lymphoid cell group 2 cells, NK cells and NK T cells (114). De Lange-Brokaar et al. reviewed immune cells, their cytokines and synovial inflammation in OA and concluded that most of immune cells found in OA synovial tissues were mast cells, macrophages and T cells while B cells, NK cells and plasma cells were detected in lower amounts; in addition, cytokines related to T cells or macrophages were detected abundantly in OA synovial tissues, indicating that T cells and macrophages were activated in OA synovial tissues (115). To further investigate the immune cells and related inflammation in OA, Klein-Wieringa et al. found that the main immune cells in the synovial tissues of OA patients were T cells and macrophages, followed by mast cells, and most of proinflammatory cytokines were produced by T cells and macrophages even without additional stimulation, among which CD4+ and CD69+ T cells were highly present (116). In addition, the amount of CD4+ T cells in synovial tissues was significantly associated with poor pain feelings classified by the visual analog scale (VAS) (116). Taken together, adaptive immune cells, particularly T cells, play a vital role in the modulation of the intestinal microbiome and osteoarthritis, and the related mechanisms are summarized in **Figure 3**.

### 4.1 T cells in the gut microbiota and OA

Among adaptive immune cells, the predominant immune cells are T cells, which are crucial in the progression of OA. To date, it has been widely accepted that significant alterations in Th17 cells, Th9 cells, T memory cells, cytotoxic T cells, regulatory T (Treg) cells and Th1 cells are found in the synovial fluid, synovial tissues and peripheral blood of people with OA (124). However, the role of follicular helper T (TFH) cells, Th22 cells and Th2 cells in the pathogenesis of OA is still uncertain.

The gut microbiota, as a risk factor for OA, has been proposed as a regulator of the T-cell response, especially for Th17 cells (125). Th17 cells, the most primitive subset of CD4+ T cells, are characterized by the production of proinflammatory cytokines, such as IL-22, IL-21 and IL-17, and proinflammatory cytokines, including IL-21, IL-6 and TGF-β, can initiate the induction of Th17 cells (118). TGF-β has a dual role in immune modulation, and low amounts of TGF-β induce the differentiation of Th17 cells whereas high amounts of TGF-β induce Treg cells to inhibit inflammation (118). Imbalances in the intestinal flora can modulate the differentiation of CD4+ T cells into Treg cells or effector T cells, which is crucial for immune homeostasis and joint inflammation (23). Segmented filamentous bacteria (SFB), which accumulate in the synovial fluids and synovium of subjects with OA, can induce Th17 cell accumulation by increasing local serum amyloid A (SAA) production, and increased SAA can stimulate DCs in the lamina propria to induce Th17 cells (23). Clostridia is a predominant type of commensal microorganism in the colon and can induce local Treg cells in the colon to inhibit inflammatory and allergic responses, which presumably are induced by DCs (126). Moradi et al. showed that abundant Treg cells accumulated in synovial fluid and the synovial membrane from individuals with OA and that they existed as activated effector memory cells (CD62L^−^CD69^+^), while fewer Treg cells were detected in peripheral blood concurrently with resting central memory cells (CD62L^+^CD69^−^) (127). So et al. showed that coadministration of Lactobacillus casei with type II collagen/glucosamine reduced arthritic alterations and suppressed cartilage destruction in an OA model; furthermore, cotreatment of Lactobacillus casei with type II collagen/glucosamine reduced proinflammatory cytokines, such as TNF-α, IFN-γ, IL-6, IL-2 and IL-1β, in CD4+ T cells (128). Jhun and colleagues found that the combination of L. acidophilus, vitamin B, and curcumin suppressed Th17 cell differentiation and maintained the Treg cell population by phosphorylated signal transducer and activator of transcription 3 (STAT3); at the same time, the combination suppressed the proinflammatory Th17-related cytokine IL-17 and increased the anti-inflammatory Treg-related cytokine IL-10 in human peripheral blood mononuclear cells (117). In a rat OA model, the combination of L. acidophilus, vitamin B, and curcumin alleviated pain, protected cartilage, regulated the anabolic/catabolic balance and reduced proinflammatory cytokines, including MCP-1, TNF-α, IL-17 and IL-1β. TFH cells can regulate the activation of B cells to generate immunoglobulins via the secretion of IL-21, and chemokine (C-X-C motif) receptor 5 (CXCR5)+CD4+ T cells are thought to be TFH cells with surface markers, such as the transcription factor, CD40 ligand, programmed death-1, inducible costimulator and CXCR5 (129). Shan et al. showed that, in comparison with healthy individuals, IL-21+ TFH cells were significantly higher in people with stage IV OA in comparison to those with stage II and stage III, and the levels of serum IFN-γ, IL-17A and IL-21 were increased concurrently (130). Furthermore, IL-21+ TFH cells were positively correlated with the level of serum CRP and WOMAC scores of OA patients. Recently, Cheng et al. found that primary bile acid, a metabolite of the gut microbiota, can inhibit the activation of CXCR5+CD4+ TFH cells (119). Butyrate, which is critical for the maintenance of intestinal microbiome homeostasis, can promote the generation of inducible Treg (iTreg) cells by enhancing histone acetylation for gene expression by inhibiting HDAC; in addition, in a carnitine palmitoyl transferase 1A (CPT1A)-dependent manner, butyrate can also facilitate iTreg differentiation by increasing fatty acid oxidation (122). In a rheumatoid arthritis model, butyrate significantly increased systemic Treg cells and reduced Th17 cells by inhibiting the expression of proinflammatory cytokines, including IL-17A, IL-1β and IL-6, and promoting anti-inflammatory IL-10 expression (131), and it is suggested that butyrate may have the same effects in OA. Campbell et al. revealed that 3β-hydroxydeoxycholic acid (isoDCA), a kind of secondary BA, can enhance the induction of colonic Treg cells by acting on DCs in a conserved noncoding sequence 1 (CNS1)-dependent manner (120). Overall, more studies are needed to uncover the role of TFH cells, Treg cells and Th17 cells in the intestinal microbiome and OA, and SCFAs can modulate Treg cell levels and Th17 cell differentiation, which can be novel targets for future management of OA.

### 4.2 B cells in the gut microbiota and OA

B cells are known to generate immunoglobulins and to regulate immunity. As immunosuppressive cells, regulatory B cells (Bregs) can maintain immunological tolerance via IL-10, IL-35, and TGF-β1 (132). Bregs can inhibit the activation of Th1 cells, Th17 cells and CD8+ T cells; regulate the differentiation of macrophages and DCs; and facilitate the induction of Treg cells (133–135). Studies have reported that oligoclonal B cells infiltrate the joint synovium of people with OA, indicating that an antigen-presented immune response might contribute to the progression of OA (136). Sun et al. revealed that IL-10 could induce B cells, and B cells were detected in the synovial fluid in OA. Compared with rheumatoid arthritis patients, in the joint synovium of OA patients, the frequency of IL-10+ B cells was higher, while the total number of IL-10+ cells in synovial B cells was lower (137). Phenotypical analysis showed that the IL-10+ B cells were IgM+CD27+ and they expressed more IL-10 and inhibited IFN-γ expression and the proliferation of autologous T cells via IL-10. IgM+CD27+ B cells in the synovial fluid were negatively associated with the severity of OA (137). Rosser et al. reported that SCFAs were reduced in rheumatoid arthritis patients and arthritic mice in comparison with healthy people and in mice, and butyrate supplementation significantly alleviated the arthritis degree (138). Supplementation with butyrate amplified AhR activation in Bregs, which relieved arthritis (138). Similarly, Yao et al. also found that SCFAs, including valerate, butyrate, propionate and acetate, were reduced in rheumatoid arthritis patients, and the amounts of butyrate, propionate and acetate were positively correlated with the frequency of Bregs instead of Tregs in the peripheral blood (139). Treatment with acetate, propionate and butyrate mitigated symptoms of arthritis, increased the frequency of Bregs and reduced the frequency of transitional B and follicular B cells in collagen-induced mouse arthritis via the FFA2 receptor (139). Similarly, Zou et al. reported that treatment with SCFAs could upregulate Bregs and ameliorate clinical scores of arthritis in collagen-induced mouse arthritis in a manner dependent on HDAC inhibitory activity (123). Taken together, the immunosuppressive role of Bregs in rheumatoid arthritis and autoimmune arthritis suggest that the gut microbiota may regulate Bregs in OA in a similar way as described above. Bregs may be a novel therapeutic choice for OA, and more *in vivo* and *in vitro* studies are needed.

## 5 Gut microbiome modulation as a new choice for OA treatment

**Figure 4:**
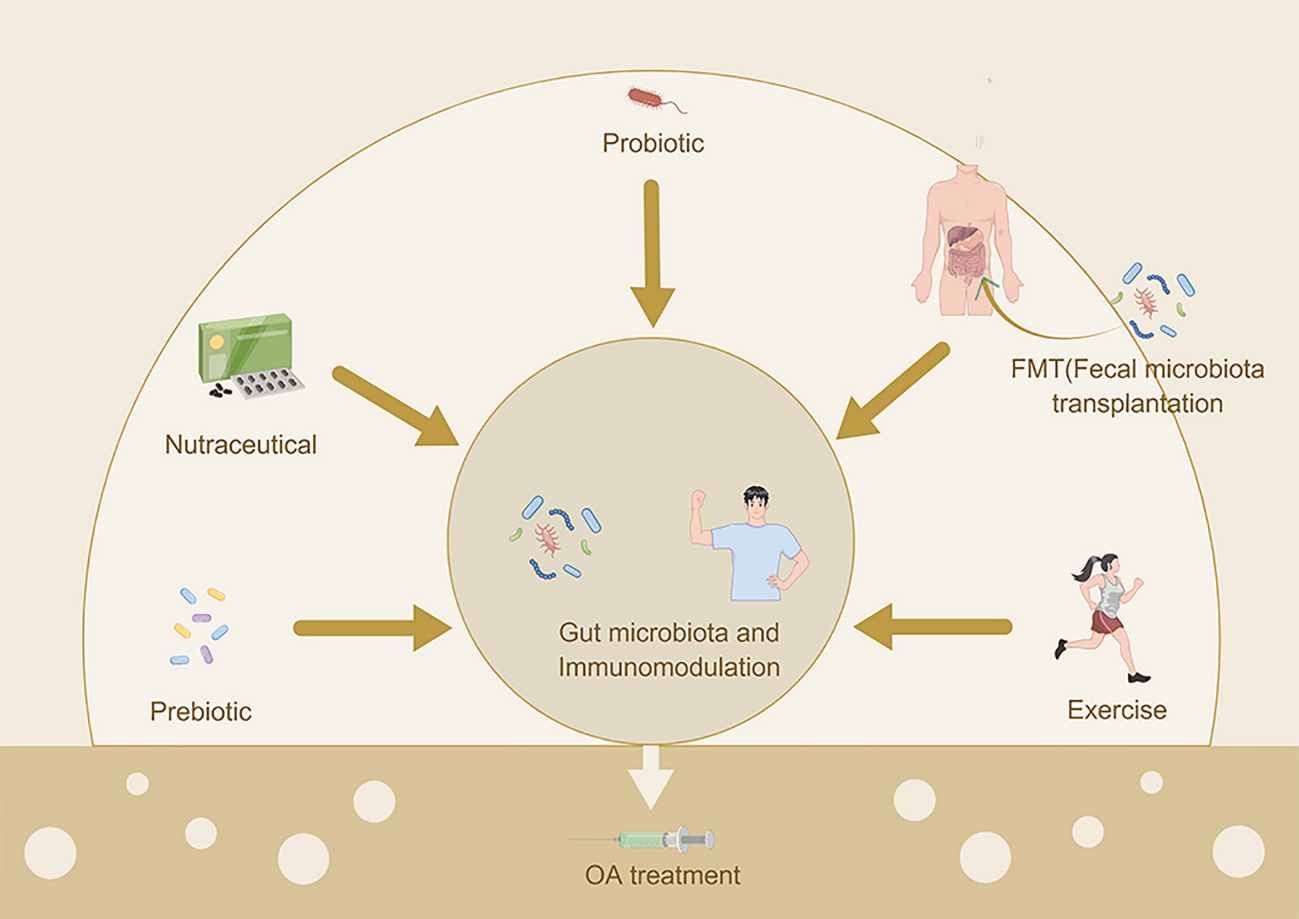
Therapeutic strategies for gut microbiota dysbiosis in OA patients.

The main characteristics of OA are chronic and progressive joint cartilage destruction and osteophyte proliferation, which ultimately result in chronic disability and a crucial burden on health care systems globally. To date, the most recognized clinical therapy for OA is palliative treatment, which ameliorates the symptoms of OA (140). Presently, many factors have been reported to act in the initiation and pathogenesis of OA; however, the intestinal microbiome is considered to be a novel pathogenic factor for osteoarthritis, and related mechanisms have been elucidated (67). Furthermore, some studies have suggested that modulation of the gut microbiota can be a new treatment for OA. Thus far, interventions consisting of prebiotics, probiotics, diet, nutraceuticals, exercise and fecal microbiota transplantation (FMT) for the intestinal microbiome in OA have been proposed (**Figure 4**).

### 5.1 Prebiotics

The selective utilization of prebiotics by the host’s gut microbiome is good for health, and the major components of prebiotics are part of carbohydrate groups, mostly oligosaccharides; however, prebiotics are not limited to carbohydrates only (141). According to the study by Schott et al., administration of oligofructose, a kind of prebiotic, restored the imbalance in the intestinal microflora in obese mice by increasing key commensal microflora, particularly beneficial Bifidobacteria, suppressed the downstream inflammatory responses in the local and systemic circulation and eventually alleviated OA-related joint cartilage injury (72). Oligofructose supplementation altered microbial populations in the gut of both lean and obese mice, probably due to the phylum Actinobacteria. The obese mice showed a reduced Bacteroidetes/Firmicutes ratio and a lack of Actinobacteria, while oligofructose could partially change the Bacteroidetes/Firmicutes ratio and increase the amount of Actinobacteria in obese mice, which is primarily associated with the abundance of Bifidobacteria. Oligofructose supplementation can induce Bifidobacteria to decrease gut permeability, increase the expression of tight junction proteins and inhibit inflammation. In addition, oligofructose reduced proinflammatory cytokines, such as MCP-1 and IL-12, and increased the anti-inflammatory cytokine IL-10 levels in obese mice. Additionally, oligofructose suppressed the migration of obesity-related macrophages to the joint synovium and reduced the proinflammatory cytokine MCP-1 levels in obese mouse joints; however, the number of tissue macrophages was not changed, indicating the role of oligofructose in the control of macrophage migration in the pathogenesis of OA. Similarly, Rios et al. observed that early intervention with oligofructose supplementation could reverse the harmful effects of a high-fat/high-sucrose (HFS) diet on joint damage in a rat OA model and could significantly ameliorate insulin resistance, restore gut microbiota dysbiosis and decrease endotoxemia (142). After oligofructose supplementation, Rios and colleagues observed an expansion of Bifidobacterium, Bacteroides/Prevotella and Roseburia and a decrease in the abundances of Akkermansia muciniphila, Methanobrevibacter, Faecalibacterium prausnitzii, Clostridium cluster I and Clostridium cluster IV. Bifidobacterium, Bacteroides/Prevotella and Roseburia were positively associated with cartilage protection, while Akkermansia muciniphila, Faecalibacterium prausnitzii, and Clostridium cluster IV were positively associated with cartilage damage. However, for rats with 12 weeks of exposure to a HFS diet, prebiotics plus exercise could not reverse the already established knee damage (143). Collectively, oligofructose can play a prophylactic role in OA development; however, oligofructose cannot repair OA-induced cartilage damage. To date, no human studies have been performed, and future studies on prebiotics and OA in humans are needed to determine the appropriate time and dose of prebiotic supplementation in people with high risk factors for OA.

### 5.2 Probiotics

Probiotics, composed of live microorganisms, play a crucial role in the modulation of the intestinal microflora by enhancing the generation of antimicrobial compounds as well as immunoglobins and reducing the release of bacterial endotoxins (144, 145). Lactobacillus casei are the most studied probiotics. Lei and colleagues conducted a randomized double-blind, placebo-controlled clinical trial with a sample size of 537 individuals with OA (146), which is the most convincing evidence. The authors reported that after 6 months of Lactobacillus casei Shirota supplementation, both the VAS and WOMAC scores were significantly reduced compared with those in the placebo control group, accompanied by a significantly decreased serum level of high sensitivity C-reactive protein (hs-CRP), and serum hs-CRP levels were significantly associated with VAS and WOMAC scores, indicating that oral administration of Lactobacillus casei might be a new option for clinical therapy in knee osteoarthritis (146). Similarly, Sullivan et al. reported that oral Lactobacillus acidophilus consumption dramatically relieved joint pain, prevented cartilage degradation, expanded beneficial bacteria such as Akkermansia muciniphila and Lachnospiraceae, and lowered the alpha diversity in the gut compared to those in the vehicle group (147). In addition, Lactobacillus acidophilus supplementation suppressed proinflammatory cytokines, including NF-κB, TNF-α, and IL-1β, and increased anti-inflammatory IL-10, suggesting that Lactobacillus acidophilus may be a safe OA disease-modifying drug. In addition to lactic acid bacteria, Sim et al. revealed that tyndallized Clostridium butyricum could protect the knee synovium and joint cartilage, increase the weight-bearing distribution by ≥20%, and dramatically reduce the number of fibrous tissues by inhibiting inflammatory factors (IL-6, leukotriene B4, Cox 2) and MMP production and increasing anti-inflammatory cytokine INF-γ (148). Henrotin et al. reported that oral consumption of lyophilized inactivated culture from Bifidobacterium longum for 12 weeks reduced the structural damage of joint cartilage, synovial inflammation and type II collagen degradation in spontaneous OA pig model, indicating a potential preventive role of Bifidobacterium longum in the progression of OA (149). According to the research by Li et al., oral supplementation with Clostridium butyricum could relieve OA pain, ameliorate cartilage damage and inhibit synovial hyperplasia by downregulating TNF-α and IL-1β in OA cartilage and synovium of rat models (150). Lin and colleagues revealed that Lactobacillus plantarum improved joint mechanical load, alleviated cartilage damage, and reduced synovial inflammation by inhibiting inflammatory factors (TNF-α, IL-1β) in OA cartilage and synovium of the anterior cruciate ligament transection-induced rat models (151). Streptococcus thermophilus (TCI633), a newly discovered bacterium in human milk, can produce hyaluronate in the gastrointestinal tract and widely reduce the inflammatory responses of synovial tissues and joint cartilage structural lesions in a dose-dependent manner in rat models. In addition, TCI633 can efficiently increase the production of type II collagen and decrease the apoptosis of chondrocytes in cartilage (152). Furthermore, Lyu et al. conducted a clinical RCT with 80 individuals for 12 weeks, and they observed no significant improvements in serum C-reactive protein and serum collagen type II C-telopeptide (sCTX-II) between the TCI633 group and the control group, which may be influenced by Kellgren/Lawrence grading, small sample size and a short observational time; however, the distinct WOMAC scores in the TCI633 group indicated that TCI633 might slow the pathological process of knee OA (153). In addition to cartilage protection, Taye et al. conducted an N-of-1 trial and showed that probiotic intervention was associated with lower pain scores, suggesting the role of probiotics in pain relief in OA patients (154).

So et al. revealed that coadministration of Lactobacillus casei and type II collagen/glucosamine could significantly relieve the pain of OA and reduce cartilage destruction in a rat OA model compared to type II collagen/glucosamine or Lactobacillus casei alone; furthermore, oral supplementation with Lactobacillus casei and type II collagen/glucosamine could significantly inhibit proinflammatory cytokines, such as TNF-α, IFN-γ, IL-17A, IL-12B, IL-12A, IL-6, IL-2, and IL-1β, and upregulate anti-inflammatory cytokines, including TGF-β, IL-10, and IL-4, in CD4+ T cells (128). In addition, administration of Lactobacillus casei and type II collagen/glucosamine significantly decreased inflammatory molecules, such as Cox-2, TNF-α, IL-6 and IL-1β, and MMPs, in synovial fibroblasts and chondrocytes in comparison with type II collagen/glucosamine or Lactobacillus casei alone. Korotkyi et al. performed an animal test to verify the effect of a multistrain probiotic with or without chondroitin sulfate addition on the expression of Col2a1, Tgfb1 and Ptgs2 in a monoiodoacetate-induced rat OA model (155). In a rat OA model, the expression of the proinflammatory cytokines Tgfb1 and Ptgs2 was upregulated, while the expression of the anti-inflammatory cytokine Col2a1 was downregulated. Separate supplementation with probiotics or chondroitin sulfate significantly suppressed the expression of Tgfb1 and Ptgs2 and increased Col2a1 expression. Compared with separate application, application of both showed significantly more alterations in the expression of Col2a1, Tgfb1 and Ptgs2 (155). In addition, Korotkyi et al. discovered that the separate application of probiotics and chondroitin sulfate slightly reduced OA scores, limited chondrocyte death and decreased subchondral bone resorption in rats; however, no significant decreases in the expression of TNF-α, NF-κB, TLR 2 and TLR 4 were detected (156). Combined treatment with probiotics and chondroitin sulfate significantly reduced OA scores and decreased the expression of TNF-α, NF-κB, TLR 2 and TLR 4 in synovial cells and chondrocytes (156). Korotkyi et al. conducted further investigation on the efficiency of probiotics and chondroitin sulfate separately or in combination in OA and observed that chondroitin sulfate and multistrain probiotics could regulate the NF-κB inflammatory signalling pathway mediated by TLR 2/4 and reduce the metabolism of cartilage, indicating that probiotics amplify the beneficial role of chondroitin sulfate in attenuating osteoarthritis (157). Wang et al. revealed that Akkermansia muciniphila, a gut commensal probiotic bacterial species, determined the role of chondroitin sulfate in OA (158). An optimum level of Akkermansia muciniphila can thicken the intestinal mucosa and activate mucosal immunity to prevent pathogen infiltration, while an overabundance of Akkermansia muciniphila can cause mucin degradation, damage to the colonic mucosa and severe leakage of endotoxin. Akkermansia muciniphila can compete with sulfatase-secreting bacteria and sulfate-reducing bacteria, which are expanded by oral chondroitin sulfate in the distal gut. In the presence of optimum levels of Akkermansia muciniphila, chondroitin sulfate can ameliorate OA; otherwise, chondroitin sulfate can aggravate OA (158). Therefore, the role of chondroitin sulfate in OA is affected by the gut microbiome, indicating that elderly patients or immunosuppressed patients may not benefit from oral chondroitin sulfate, and the combination of chondroitin sulfate with probiotics may show better improvements in OA patients. Jhun and colleagues found that the combination of L. acidophilus, vitamin B, and curcumin suppressed Th17 cell differentiation and maintained the Treg cell population by phosphorylating STAT3; at the same time, the combination suppressed the proinflammatory Th17-related cytokine IL-17 level and increased the anti-inflammatory Treg-related cytokine IL-10 level in human peripheral blood mononuclear cells (117). Moreover, the combination of L. acidophilus, vitamin B, and curcumin alleviated pain, preserved joint cartilage, increased the anabolic enzyme TIMP13, decreased the catabolic enzyme MMP13 and reduced the levels of proinflammatory cytokines, such as MCP-1, TNF-α, IL-17 and IL-1β, in the monosodium iodoacetate-induced rat OA model. In addition, the combination upregulated TIMP13 and downregulated MMP13 in human chondrocytes. The combination of probiotic complex (multiple Lactobacillus, Bifdobacterium, Streptococcus species), rosavin plus zinc exhibited anti-inflammatory and antioxidant properties, relieved pain, and improved cartilage destruction by inhibiting the production of catabolic factors (MMP3, TIMP3) as well as proinflammatory cytokines (TNF-α, IL-6) and increasing the anti-inflammatory cytokine IL-10 in rat models (159).

In sum, probiotics can mitigate the development of OA by relieving pain, inhibiting inflammatory responses and restoring the imbalance in the intestinal microbiome. In addition, the synergistic effect of probiotics and other pharmaceuticals on the gut microbiota and OA is better than their separate application. To better treat OA, large sample size clinical studies of probiotics and OA are necessary.

### 5.3 Nutraceuticals

Glucosamine sulfate and chondroitin sulfate are common medicines used to relieve the symptoms of OA in clinical therapy and are recommended by the European Society for Clinical and Economic Aspects of Osteoporosis, Osteoarthritis and Musculoskeletal Diseases; in contrast, chondroitin sulfate and glucosamine sulfate are not recommended for OA by the Osteoarthritis Research Society International (160). The intestinal microbiome can affect the metabolism of glucosamine sulfate and chondroitin sulfate in the gastrointestinal tract, so oral administration of glucosamine sulfate and chondroitin sulfate is limited. By analyzing the degradation products of CSA (the main component of chondroitin sulfate pharmakon) of the intestinal microbiome from six healthy humans, Shang et al. observed that each subject’s microflora exhibited different degrading activities, but in all cases, the end products contained ΔUA-GalNAc4S, suggesting that chondroitin sulfate could be easily degraded to various degrees by many microbial communities, which may contribute to the poor bioavailability and different effects of chondroitin sulfate during the management of OA patients (161). In addition, Liu et al. investigated the impact of chondroitin sulfate disaccharides on the content and function of the intestinal microflora in mice under stressed conditions and in healthy mice. They found that chondroitin sulfate disaccharides can reduce blood LPS levels and increase the levels of fecal total SCFAs, especially butyrate. Additionally, chondroitin sulfate supplementation reduced the Proteobacteria abundance and increased that of Bacteroidetes in the gut, suggesting that chondroitin sulfate is a bioactive nutraceutical that inhibits inflammation and protects the gut (162). As we mentioned above, chondroitin sulfate can ameliorate OA with the assistance of Akkermansia muciniphila (158). In combination with beneficial gut microbiota, chondroitin sulfate can significantly decrease the expression of proinflammatory cytokines, such as Ptgs2 and Tgfb1, and increase proinflammatory Col2a1 expression in rat OA models (155). Recently, Zhang et al. investigated the effect of type II collagen peptides, cartilage powder from chicken and chondroitin sulfate in rat OA models and showed that chondroitin sulfate exhibited the best therapeutic effect for OA, as it dramatically improved joint cartilage morphology and decreased OA scores by reducing inflammatory cytokine levels in the serum or synovial fluid, such as prostaglandin E2, TNF-α, IL-1β, IL-6, and IL-17, and increasing the abundance of Bacteroidetes (163). For glucosamine, Sicard et al. reported that the mucin sugar N-acetyl-glucosamine could decrease biofilm formation of Escherichia coli by influencing the virulence properties of pathogenic E. coli, which help maintain the gut barrier (164). According to the study by Coulson et al., oral administration of glucosamine sulfate or green lipped mussel extract can improve symptoms of OA via the regulation of the components, metabolism and immunity of the intestinal microbiome; notably, the decrease in Clostridia was associated with improvements in the symptoms of OA, and Clostridia can induce the generation of Th17 cells to inhibit inflammation (165). Shmagel et al. performed a systematic review on the effects of glucosamine sulfate or chondroitin sulfate on intestinal microbial composition in humans and animals and reported that chondroitin sulfate could increase the amount of the genus Bacteroides in human and mouse intestines, whereas evidence of glucosamine sulfate on the gut microbiome was limited (166). Pycnogenol, a proprietary extract from pine bark, can be metabolized by the intestinal microbiome and alleviate the symptoms of OA via anti-inflammatory, antioxidative and chondroprotective effects. In addition, oral administration of pycnogenol could reduce gastrointestinal complications and hospitalization of people with OA by sparing the use of nonsteroidal anti-inflammatory drugs (167). In summary, nutraceuticals can modulate the gut microbiota and mitigate the symptoms of OA, whereas nutraceuticals can be affected in the gut by the intestinal microbiome. More studies on nutraceuticals are warranted for them to serve as a novel option for OA treatment.

### 5.4 Exercise

Exercise has been proven to alleviate pain and ameliorate joint function in OA patients (168). In addition to the reduction in mechanical load on subchondral bone and cartilage, exercises can increase the amount of beneficial microbial species, enrich the diversity of the microflora and improve the development of symbiotic bacteria, therefore providing benefits to the host (169). Some studies have reported that physical exercise impacts the content of the intestinal microbiome, such as increasing the ratio of Bacteroidetes-Firmicutes, enhancing the immune function of the intestinal mucosa, improving the profile of BAs and increasing the generation of SCFAs, such as butyrate, acetate and propionate (170), which are beneficial for mitigating symptoms in OA patients. As mentioned above, interventions with aerobic exercise and prebiotic fiber separately or in combination significantly protected knee joints from damage in rat OA models, suggesting that exercise may influence the progression of OA in coordination with the gut microbiota (142). To further interpret the significant synergistic effect of nutraceuticals and exercise in dealing with osteoarthritis, de Sire et al. summarized that synergistic physiogenomic and nutrigenomic treatment could reduce and slow down the complicated pathological characteristics of OA through apoptotic, proinflammatory and anti-inflammatory signals (171). Recently, Li et al. reported that wheel-running exercise enriched the diversity of the intestinal microbiome, modified the intestinal microbiome, reduced the amount of LPS in synovial fluids and blood, reduced the expression of MMP-13 and TLR4 and ameliorated cartilage damage in rat OA models, suggesting that moderate exercise is a novel therapeutic option for preventing and treating obesity-related OA (172). Whole body vibration (WBV), a novel kind of neuromuscular technique, uses the vibration generated by a vibration platform to improve the bioactivity of muscle groups. According to the research by Yu et al., M1 macrophages were polarized to M2 macrophages through the induction of WBV, and WBV could alter the fecal microbiome in diabetic mice (173). In addition, Song et al. reported novel impacts of WBV on the gut microbiome and immunity (174). They observed significantly increased levels of CD4 and CD25 positive lymphocytes and enhanced differentiation of Treg cells in the WBV group. Further microbiome analysis revealed that the amount of Lactobacillus animalis was dramatically elevated as a result of vibration application in mice, while Lactobacillus sanfranciscensis and Lactobacillus paraplantarum were increased in humans. Moreover, Lactobacillus spp. was associated with Treg cell differentiation in humans and mice (174). In addition, studies on the application of WBV in knee OA have been summarized. Furthermore, Wang et al. summarized possible mechanisms of WBV in knee OA, such as bone microstructure improvement, joint cartilage degeneration delay and the modulation of inflammatory responses, which provide ideas for the future application of WBV in the management of knee OA (174). To date, studies regarding exercise, the gut microbiota and OA are scarce, and more relevant studies are warranted to interpret the precise mechanisms and guide the application of exercise in OA treatment.

### 5.5 Fecal microbiota transplantation

To address diseases correlated with the intestinal microbiome, fecal microbiota transplantation (FMT) has been introduced, where the nature of FMT is to transfer a healthy subject’s feces into another’s distal gastrointestinal tract (175). Notably, during the treatment for recurrent Clostridium difficile infection, FMT has been proven to be a dramatic success, and a relevant standard of fecal preparation for FMT was reported (176). Given its excellent manifestation in eradicating Clostridium difficile infection, researchers have explored the application of FMT in some diseases, including allergic diseases, autoimmune diseases, neurodevelopmental disorders, metabolic syndrome, irritable bowel syndrome and inflammatory disease (177), and after a trial in inflammatory bowel disease, FMT was considered to be a possible therapeutic option (178). As mentioned above, the gut microbiota is considered a virtual organ with 4 broad functions, namely, playing roles in nutrition and metabolism, epithelial cell differentiation and proliferation, immunomodulation and pathogenic resistance and clearance (12). Several studies have revealed that enteric dysbacteriosis was linked to the onset and development of OA; moreover, FMT was considered to be an important method in the manipulation of gut dysbiosis (179); hence, the hypothesis of FMT application in OA has been proposed. Huang et al. conducted a clinical trial to transfer human fecal samples from a group with knee osteoarthritis with metabolic syndrome, a group with knee osteoarthritis without metabolic syndrome and a healthy control group into germ-free mice 2 weeks prior to surgically induced OA by meniscal/ligamentous injury (20). In germ-free mice without transplantsation, minimal signs of OA and synovitis were observed. In mice transplanted with fecal samples from subjects with knee osteoarthritis with metabolic syndrome, Huang et al. found higher cartilage damage scores, higher levels of serum inflammatory factors (IL-1β, IL-6 and macrophage inflammatory protein-1α), higher serum LPS levels, higher intestinal permeability and a lower α diversity of the gut microbiome. By analyzing the correlation between the gut bacterial genera, cartilage histology scores and inflammatory factors, the enrichment of Fusobacterium and Faecalibacterium and the reduced abundance of Ruminococcaceae were significantly associated with both higher cartilage histology scores and greater inflammatory factor levels, indicating the direct gut microbiome-knee osteoarthritis connection and the possibility of gut microbiome targeted intervention. Given the modification of FMT in the intestinal microbiome, the application of FMT for OA treatment is possible. However, relevant studies are rare, and problems including donor selection and filtration, standards of fecal preparation, indications and contraindications and recipient monitoring are still need to be taken into consideration.

## 6 Conclusions and perspectives

Currently, the incidence rate and disability rate of OA are very high, which makes OA a crucial public health problem globally. The pathogenesis of OA needs to be fully elucidated to develop effective means for preventing and treating OA. The gut microbiome is one of the risk factors correlated with OA; however, the causal effect of the gut microbiome and OA is still controversial. As mentioned above, the gut microbiome interacts with risk factors for OA, such as obesity, estrogen, age, sex, diet, metabolic syndrome, genetics, inflammation, mechanical loading and exercise. In this manuscript, we have summarized the potential immune mechanisms of the intestinal microbiome during the onset and progression of knee osteoarthritis and relevant studies on the immunomodulation of the gut microbiome in the management of OA. To investigate the pathogenesis and uncover the immune mechanisms between OA and the gut microbiota, more research on the distribution and composition of the intestinal microbiome and immune cells in synovial fluids and synovial tissues is warranted. Moreover, the alterations in intestinal microflora in individuals with OA need further investigation to identify the specific pathogens involved in the immune responses. In addition, more research is required to identify the potential common pathways and the synergistic effect between prebiotics, probiotics, nutraceuticals and exercise in the immune modulation of the intestinal microflora. Due to the enormous development of metabolomics, transcriptomics and next-generation sequencing, future studies should include more novel interventions on immune cell modifications and gene regulation on specific gut microbiota related to OA, and it is likely that more studies will uncover the relationship between distinct cell subgroups and the gut microbiota in OA. Additionally, signals associated with enteric dysbacteriosis and immune modulation in OA patients will be identified, and novel therapies targeting the gut microbiota and immunomodulation will be proposed to prevent the progression of OA.

## Author contributions

CS and JM conceived the study. CS, XZ, and TG screened the literatures and wrote the review. JM reviewed and edited the manuscript. All authors contributed to the article and approved the submitted version.
